# Characteristics of patients with advanced cancer preferring not to know prognosis: a multicenter survey study

**DOI:** 10.1186/s12885-022-09911-8

**Published:** 2022-09-01

**Authors:** Naomi C. A. van der Velden, Hanneke W. M. van Laarhoven, Sjaak A. Burgers, Lizza E. L. Hendriks, Filip Y. F. L. de Vos, Anne-Marie C. Dingemans, Joost Jansen, Jan-Maarten W. van Haarst, Joyce Dits, Ellen MA Smets, Inge Henselmans

**Affiliations:** 1 Department of Medical Psychology, Amsterdam Public Health Research Institute, Cancer Center Amsterdam, Amsterdam University Medical Centers, University of Amsterdam, Meibergdreef 9, 1105 AZ Amsterdam, The Netherlands; 2 Department of Medical Oncology, Cancer Center Amsterdam, Amsterdam University Medical Centers, University of Amsterdam, Amsterdam, The Netherlands; 3 Department of Thoracic Oncology, Netherlands Cancer Institute, Antoni Van Leeuwenhoek Ziekenhuis, Amsterdam, The Netherlands; 4 Department of Pulmonary Diseases, GROW School for Oncology and Developmental Biology, Maastricht University Medical Center+, Maastricht, The Netherlands; 5 Department of Medical Oncology, University Medical Center Utrecht, Utrecht University, Utrecht, The Netherlands; 6 Department of Pulmonary Diseases, Erasmus MC Cancer Institute, Erasmus University Medical Center, Rotterdam, The Netherlands; 7 Department of Respiratory Medicine and Department of Surgery, Tergooi Ziekenhuis, Hilversum, The Netherlands; 8 Department of Pulmonology, Franciscus Gasthuis en Vlietland, Rotterdam, The Netherlands

**Keywords:** Patient Preference, Prognosis, Physician–patient relations, Communication, Disclosure, Palliative care, Neoplasm metastasis, Cross-sectional studies

## Abstract

**Background:**

For some patients with advanced cancer not knowing prognosis is essential. Yet, in an era of informed decision-making, the potential protective function of unawareness is easily overlooked. We aimed to investigate 1) the proportion of advanced cancer patients preferring not to know prognosis; 2) the reasons underlying patients’ prognostic information preference; 3) the characteristics associated with patients’ prognostic information preference; and 4) the concordance between physicians’ perceived and patients’ actual prognostic information preference.

**Methods:**

This is a cross-sectional study with structured surveys (PROSPECT). Medical and thoracic oncologists included patients (*n* = 524), from seven Dutch hospitals, with metastatic/inoperable cancer and an expected median overall survival of ≤ 12 months. For analysis, descriptive statistics and logistic regression models were used.

**Results:**

Twenty-five to 31% of patients preferred not to know a general life expectancy estimate or the 5/2/1-year mortality risk. Compared to patients preferring to know prognosis, patients preferring unawareness more often reported optimism, avoidance and inability to comprehend information as reasons for wanting *limited* information; and less often reported expectations of others, anxiety, autonomy and a sense of control as reasons for wanting *complete* information. Females (*p* < .05), patients receiving a further line of systemic treatment (*p* < .01) and patients with strong fighting spirit (*p* < .001) were more likely to prefer not to know prognosis. Concordance between physicians’ perceived and patients’ actual prognostic information preference was poor (kappa = 0.07).

**Conclusions:**

We encourage physicians to explore patients’ prognostic information preferences and the underlying reasons explicitly, enabling individually tailored communication. Future studies may investigate changes in patients’ prognostic information preferences over time and examine the impact of prognostic disclosure on patients who prefer unawareness.

**Supplementary Information:**

The online version contains supplementary material available at 10.1186/s12885-022-09911-8.

## Background

Communication of prognosis is important for decision-making in palliative cancer care. It enables patients with advanced cancer to weigh the risks and benefits of treatment, form future care plans and prepare for the end-of-life [1–9]. Still, prognostic unawareness is common [2, 10–13]. Prognostic unawareness could relate to physicians’ communication, yet it might also correspond to the approximately 20% of patients preferring not to know prognosis [9, 13–25]. In an era of informed decision-making, the protective function of ignorance is easily overlooked [26].

Not knowing prognosis may be essential for patients as it allows hope [15, 16, 27–31]. Other patients may avoid prognostic estimates out of fear that these evoke negative emotions or become reality [14, 32–36]. Some patients find prognosis too uncertain and therefore useless [9], or feel relieved when not having to understand medical information [37]. Cultural considerations could also motivate patients’ wish not to know prognosis [9, 34, 37].

Patients’ reasons for preferring prognostic unawareness may be rooted in personal characteristics. Individuals rejecting prognosis to maintain hope might have an optimistic personality and strong fighting spirit [38, 39]. An anxious personality and avoidant coping style could predispose patients’ wish to avoid frightful information [30]. Intolerance for uncertainty may underlie patients’ aversion to unsure predictions, and limited numeracy skills might explain perceived inability to understand prognostic information [38]. Perhaps, trusting patients prefer to rely on the physician rather than seeking information [40]. Still, relations between patients’ prognostic information preference and personal characteristics remain understudied. Besides, while some studies reported associations with older age, female sex and lower income, research investigating the background and clinical characteristics of patients who reject prognosis is scarce [32, 34, 41–44].

Literature indicates that oncologists uncommonly explore patients’ information preferences and poorly tailor information [45, 46]. Few studies investigated oncologists’ ability to estimate patients’ information needs, yet suggest that physicians struggle with judging individuals’ prognostic information preferences [25, 47]. This could be problematic, as prognostic non-disclosure impedes decision-making among patients who want information, whereas disclosing prognostic estimates to patients preferring unawareness may cause psychological harm [48].

Thus far, literature has focused on improving prognostic disclosure (e.g., guidelines, training, question prompt lists). Insight into the characteristics and reasons of patients with a preference not to know prognosis, and physicians’ knowledge hereof, is necessary to promote tailored communication. Hence, we aimed to investigate the 1) proportion of patients with advanced cancer preferring not to know prognosis; 2) reasons underlying patients’ prognostic information preference; 3) characteristics associated with patients’ prognostic information preference; and 4) concordance between physicians’ perceived and patients’ actual prognostic information preference. For the first aim, we distinguished between a life expectancy estimate (i.e., median overall survival) and the 5-, 2- and 1-year mortality risk. For the subsequent aims, we used the 1-year mortality risk, given its importance for informed decision-making and end-of-life preparation among patients with advanced cancer [49].

## Methods

### Study design

We conducted a cross-sectional survey study about prognostic information preferences and prognostic awareness among patients with advanced cancer, caregivers and physicians in the Netherlands (PROSPECT: Understanding Prognosis in Palliative Cancer Care, September 2019 – June 2021). For this paper, we used patients’ prognostic information preferences as the primary outcomes and excluded caregiver data. This report adheres to the STROBE criteria [50].

### Sample and procedure

Medical oncologists and thoracic oncologists (in training) affiliated with seven (non)academic hospitals were invited. Consenting physicians screened patients, whom they had seen at least once, for eligibility consecutively. Eligible patients were ≥ 18 years, had Dutch language proficiency, had an incurable metastatic/inoperable tumor and had an estimated median overall survival of ≤ 12 months at group-level (at diagnosis of advanced disease or after disease progression). Additional file 1 shows eligible tumor types, including treatment type and line. Patients were informed about the study’s focus in general terms (i.e., patients’ views on illness, treatment and prospects), yet blinded to the prognostic eligibility criteria. Physicians and patients provided written informed consent and participated online or on paper. Procedures complied with the Helsinki Declaration. All institutional and local medical ethics review boards provided exemption from formal approval.

We performed a priori power calculations (α-level = 0.05, power = 0.80, Cohen’s *d* = 0.5) to establish differences in patient characteristics (mostly continuous) between patients with and without a preference for prognostic information. We assumed that ≥ 20% of patients preferred prognostic unawareness based on literature [14, 22, 23]. Supposing that physicians play a minimal role in patients’ information preferences, we adopted an average cluster size of 10 (patients per physician) and an intraclass correlation of 0.05. The required sample size for the current analyses comprised 331 patients. However, the PROSPECT study was set out to answer multiple research questions, of which some required a larger sample size. We included a sample of > 331 patients to reach sufficient power for the entire study (see Additional file 2 for configuration of the PROSPECT sample).

### Measures

#### Patients’ prognostic information preferences and underlying reasons

We measured patients’ preference to know a life expectancy estimate and the 5/2/1-year mortality risk on a binary scale (yes/no) with four adjusted items [22]: “Are you a person who wants to know…” followed by, for example, “the likelihood of dying from your cancer within one year from now?”.

We used the 26-item Considerations Concerning Cancer Information Questionnaire (CCCI) to measure patients’ reasons for wanting limited information about their disease and treatment (subscales “optimism”, “comprehension”, “not wanting to be a burden”, “avoidance”) and complete information about their disease and treatment (subscales “expectations of others”, “anxiety”, “autonomy”, “sense of control”) [38]. Items (e.g., “I don’t need to know everything because it may frighten me”) were scored (1–5, “never” to “always”) and averaged per subscale. We included reasons for wanting complete information about disease and treatment to explore all considerations that underlie patients’ information preferences and to allow comparison with patients’ agreement with reasons for wanting limited information.

#### Background characteristics

Patients reported their sex (male/female), age, education (low/medium/high), nationality (Dutch/other), religion (Christianity/other/none) and presence of children < 18 years (yes/no).

We measured health literacy with the 3-item Set of Brief Screening Questions (SBSQ-D) [51, 52]. Items (e.g., “How confident are you filling out forms by yourself?”) were scored (0–4, “not at all confident” to “extremely confident”) and averaged.

We assessed patients’ numeracy with the 8-item Subjective Numeracy Scale (SNS) [53]. Items (e.g., “How good are you at working with percentages?”) were scored (1–6, “not at all good” to “extremely good”) and averaged.

#### Clinical characteristics

Physicians reported patients’ tumor type and line of systemic treatment administered during study participation (none/first/second/ ≥ third). The category “none” included patients who might have had systemic treatment prior to study participation and/or may receive systemic treatment in the future. Additionally, patients in the category “none” could have received non-systemic treatment during study participation (e.g., radiotherapy, best supportive care), yet this was not reported. Physicians registered patients’ date of diagnosis of metastatic/inoperable cancer to calculate time since diagnosis.

We measured patients’ perceived likelihood of dying within one year with one item: “How likely is it you will die from your cancer within one year from now?” (1–7, “extremely unlikely”, “very unlikely”, “unlikely”, “possibly”, “likely”, “very likely”, “extremely likely”). The “very (un)likely” and “extremely (un)likely” categories were combined in the analyses to reduce the number of statistical comparisons.

We assessed health-related quality of life with the 2-item Global Health Status subscale (GHS) of the EORTC Quality of Life Questionnaire (EORTC-QLQ-C30) [54]. Items (i.e., “How would you rate your overall 1) health and 2) quality of life during the past week?”) were scored (1–7, “very poor” to “excellent”). Scores were transformed to a 0–100 scale.

#### Personal characteristics

We measured fighting spirit (i.e., viewing cancer as a challenge) with the 4-item fighting spirit subscale of the Mini Mental Adjustment to Cancer (mini-MAC) scale [55]. Items (e.g., “I am determined to beat this disease”) were scored (1–4, “does not apply at all to me” to “totally applies to me”) and summed.

We assessed trait optimism with the 10-item Life Orientation Test-Revised (LOT-R) [56]. Six items (e.g., “I’m always optimistic about my future”) were scored (0–4, “strongly disagree” to “strongly agree”) and summed, as the others were filler items.

We measured trait anxiety (i.e., stable aspects of proneness to anxiety) with the 20-item trait scale of the Spielberger State and Trait Anxiety Inventory (STAI) [57]. Items (e.g., “I feel nervous and restless”) were scored (1–4, “not at all” to “very much so”) and summed.

We assessed avoidance coping with the 8-item avoidance subscale of the Utrecht Coping List (UCL) [58]. Items (e.g., “Avoiding difficult situations”) were scored from (1–4, “never” to “very often”) and summed.

We measured uncertainty tolerance (i.e., perceiving ambiguous situations as desirable) [59] with the 7-item Tolerance for Ambiguity (TFA) [60]. Items (e.g., “If I am uncertain about the responsibilities involved in a particular task, I get very anxious”) were scored (1–6, “strongly agree” to “strongly disagree”) and summed.

We assessed patients’ trust in the physician with the 5-item Trust in Oncologist Scale-Short Form (TiOS-SF) [61]. Items (e.g., “All in all, you have complete trust in your doctor”) were scored (1–5, “strongly disagree” to “strongly agree”) and averaged.

#### Physicians’ perceptions of patients’ prognostic information preference

Physicians reported their perception of each patient’s prognostic information preference with an adjusted item [22]: “Is this patient a person who wants to know the likelihood of him/her dying within one year from now?”. Physicians answered: “Yes, I think so” or “No, I don’t think so”.

### Statistical analysis

We used IBM SPSS Statistics 26 for all analyses. Missing data were reported and not imputed. To present patients’ prognostic information preferences (i.e., life expectancy estimate; 5/2/1-year mortality risk), we used descriptive statistics. We described the agreement with reasons for wanting limited and complete information for patients with and without a preference to know the 1-year mortality risk separately and compared means (T-tests).

To investigate differences between patients with and without a preference to know the 1-year mortality risk in patient characteristics, we performed T-tests and Chi^2^-tests. We examined clustering of data within physicians (intraclass correlation ≥ 10%), indicating a need for multilevel analysis [62–64].

To examine if patients’ information preference regarding the 1-year mortality risk (0 = preferring to know, 1 = preferring not to know) related to patient characteristics, we constructed a logistic regression model. Intercorrelations between independent variables were calculated to identify multicollinearity (*r* > 0.80) [65]. We entered variables in the multivariate model one by one, hierarchically (i.e., background, clinical, personal). After each entry, we evaluated variables at a liberal α-level (*p* < 0.20), preventing elimination due to confounding or modification effects. We tested the resultant model with an α-level of *p* < 0.05. We eliminated non-significant variables one by one to simplify the final model.

To present the concordance between physicians’ perceived and patients’ actual information preference regarding the 1-year mortality risk, we calculated kappa values (poor, < 0.20; fair, 0.21–0.40; moderate, 0.41–0.60; good, 0.61–0.80; very good, 0.81–1.00) [66].

## Results

PROSPECT included 540 patients and/or caregivers (response rate 62%; see Additional file 2), of whom 524 patients reported their prognostic information preferences. Patients were consulted by *n* = 33 medical oncologists and *n* = 21 thoracic oncologists (*M*_included patients_ = 20). About half of patients was male; the mean age was 64 years (Table 1). Table 2 presents characteristics of patients with and without a preference to know the 1-year mortality risk.

**Table 1:** Background, clinical and personal characteristics of the total sample

Patient characteristics	Cronbach’s alpha ^a^	Total sample *n* = 524
Sex (male), % (*n*)		54.8 (287)
Age (years), mean ± SD		63.9 ± 11.0
Education, % (*n*) ^b^		
Low		37.9 (198)
Medium		26.6 (139)
High		35.4 (185)
Health literacy (SBSQ-D, 0–4), mean ± SD ^c^	.71	3.2 ± 0.8
Numeracy (SNS, 1–6), mean ± SD ^d^	.90	4.2 ± 1.2
Nationality (Dutch), % (*n*)		95.4 (500)
Religion, % (*n*)		
None		59.0 (309)
Christianity		37.0 (194)
Other ^e^		4.0 (21)
Presence of children < 18, % (*n*) ^f^		10.3 (54)
Time since diagnosis (months), mean ± SD ^g^		17.8 ± 21.5
Line of systemic treatment during study participation, % (*n*) ^h^		
None		23.6 (121)
First line		43.5 (223)
Second line		20.1 (103)
≥ Third line		12.9 (66)
Tumor type, % (*n*) ^d^		
Lung		24.1 (125)
Pleura		6.0 (31)
Oesophagogastric		13.7 (71)
Pancreatic		6.9 (36)
Other gastrointestinal		14.9 (77)
Colorectal		2.9 (15)
Brain		11.8 (61)
Gynaecological		9.5 (49)
Soft tissue		2.7 (14)
Other (each type *n* < 10) ^i^		7.5 (39)
Patients’ perceived likelihood of dying in one year, % (*n*) ^j^		
Very to extremely unlikely		24.3 (125)
Unlikely		10.5 (54)
Possibly		36.4 (187)
Likely		7.8 (40)
Very to extremely likely		21.0 (108)
Health-related quality of life (GHS, 0–100), mean ± SD ^f^	.88	63.0 ± 21.0
Fighting spirit (mini-MAC, 4–16), mean ± SD ^k^	.67	11.5 ± 2.7
Trait optimism (LOT-R, 0–24), mean ± SD ^g^	.73	14.6 ± 3.9
Trait anxiety (STAI-trait, 20–80), mean ± SD ^g^	.94	39.7 ± 10.7
Avoidance coping (UCL, 8–32), mean ± SD ^l^	.72	15.5 ± 3.3
Uncertainty tolerance (TFA, 7–42), mean ± SD ^l^	.71	25.8 ± 5.9
Trust in the physician (TiOS-SF, 1–5), mean ± SD ^g^	.92	4.3 ± 0.7

^a^Interpretation: < 0.50 unacceptable, 0.50–0.60 poor, 0.60–0.70 questionable, 0.70–0.80 acceptable, 0.80–0.90 good, 0.90–1.00 excellent

^b^*n* = 522/524 (2 missing). Low vocational education; medium level vocational education; high vocational or academic education

^c^*n* = 515/524 (9 missing)

^d^*n* = 518/524 (6 missing)

^e^Including Islam, Buddhism, Hinduism, Judaism, Humanism, spirituality and “own belief”

^f^*n* = 523/524 (1 missing)

^g^*n* = 517/524 (7 missing)

^h^*n* = 513/524 (11 missing)

^i^Including melanoma, head and neck, thyroid, breast, vagina, prostate, bladder, kidney, adrenal cortex, bone, carcinoid and unknown primary tumors

^j^*n* = 514/524 (10 missing)

^k^*n* = 511/524 (13 missing)

^l^*n* = 516/524 (8 missing)

*n* Sample size, *SD* Standard deviation, *SBSQ-D* Set of Brief Screening Questions-Dutch, *SNS* Subjective Numeracy Scale, *GHS* Global Health Status from the EORTC-QLQ-C30, *EORTC-QLQ-C30* European Organization for Research and Treatment of Cancer Quality of Life Questionnaire for Cancer, *MAC* Mental Adjustment to Cancer, *LOT-R* Life Orientation Test-Revised, *STAI* Spielberger State and Trait Anxiety Inventory, *UCL* Utrecht Coping List, *TFA* Tolerance for Ambiguity, *TiOS-SF* Trust in Oncologist Scale-Short Form

**Table 2:** Background, clinical and personal characteristics of patients with and without a preference to know prognosis

Patient characteristics	Patients preferring not to know 1-year mortality risk (30.7%) *n* = 161/524	Patients preferring to know 1-year mortality risk (69.3%) *n* = 363/524
Sex, % (*n*) **		
Male	25.4 (73) ^1^	74.6 (214)
Female	37.1 (88) ^2^	62.9 (149)
Age (years), mean ± SD	64.3 ± 10.6	63.7 ± 11.2
Education, % (*n*) ^a^ *		
Low	37.4 (74) ^1^	62.6 (124)
Medium	27.3 (38) ^1, 2^	72.7 (101)
High	26.5 (49) ^2^	73.5 (136)
Health literacy (SBSQ-D, 0–4), mean ± SD ^b^	3.2 ± 0.8	3.3 ± 0.8
Numeracy (SNS, 1–6), mean ± SD ^c^ *	4.0 ± 1.1	4.3 ± 1.2
Nationality (Dutch), % (*n*)	95.7 (154)	95.3 (346)
Religion, % (*n*)		
None	28.5 (88)	71.5 (221)
Christianity	33.0 (64)	67.0 (130)
Other ^d^	42.9 (9)	57.1 (12)
Presence of children < 18, % (*n*) ^e^		
Yes	31.5 (17)	68.5 (37)
No	32.3 (121)	67.7 (254)
Time since diagnosis (months), mean ± SD ^f^	20.3 ± 23.5	16.7 ± 20.5
Line of systemic treatment during study participation, % (*n*) ^g^ **		
None	22.3 (27) ^1^	77.7 (94)
First line	29.1 (65) ^1, 2^	70.9 (158)
Second line	38.8 (40) ^2, 3^	61.2 (63)
≥ Third line	43.9 (29) ^3^	56.1 (37)
Tumor type, % (*n*) ^c^		
Lung	31.2 (39)	68.8 (86)
Pleura	25.8 (8)	72.4 (23)
Oesophagogastric	15.5 (11)	84.5 (60)
Pancreatic	22.2 (8)	77.8 (28)
Other gastrointestinal	35.1 (27)	64.9 (50)
Colorectal	33.3 (5)	66.7 (10)
Brain	36.1 (22)	63.9 (39)
Gynaecological	40.8 (20)	59.2 (29)
Soft tissue	42.9 (6)	57.1 (8)
Other (each type *n* < 10) ^h^	38.5 (15)	61.5 (24)
Patients’ perceived likelihood of dying in one year, % (*n*) ^I^ ***		
Very to extremely unlikely	44.8 (56) ^1^	55.2 (69)
Unlikely	24.1 (13) ^2, 3^	75.9 (41)
Possibly	31.0 (58) ^3^	69.0 (129)
Likely	22.5 (9) ^2, 3^	77.5 (31)
Very to extremely likely	20.4 (22) ^2^	79.6 (86)
Health-related quality of life (GHS, 0–100), mean ± SD ^e^ **	66.7 ± 20.9	61.4 ± 20.8
Fighting spirit (mini-MAC, 4–16), mean ± SD ^j^ ***	12.3 ± 2.4	11.1 ± 2.8
Trait optimism (LOT-R, 0–24), mean ± SD ^f^	15.1 ± 4.2	14.4 ± 3.7
Trait anxiety (STAI-trait, 20–80), mean ± SD ^f^ *	38.2 ± 10.7	40.4 ± 10.6
Avoidance coping (UCL, 8–32), mean ± SD ^k^	15.8 ± 3.3	15.4 ± 3.3
Uncertainty tolerance (TFA, 7–42), mean ± SD ^k^	26.1 ± 5.9	25.7 ± 5.9
Trust in the physician (TiOS-SF, 1–5), mean ± SD ^f^	4.3 ± 0.6	4.3 ± 0.7

^1, 2, 3^ Proportions with similar superscripted numbers do not differ significantly from each other (α = .05)

^a^ *n* = 522/524 (2 missing). Low vocational education; medium level vocational education; high vocational or academic education

^b^ *n* = 515/524 (9 missing)

^c^ *n* = 518/524 (6 missing)

^d^ Including Islam, Buddhism, Hinduism, Judaism, Humanism, spirituality and “own belief”

^e^ *n* = 523/524 (1 missing)

^f^ *n* = 517/524 (7 missing)

^g^ *n* = 513/524 (11 missing)

^h^ Including melanoma, head and neck, thyroid, breast, vagina, prostate, bladder, kidney, adrenal cortex, bone, carcinoid and unknown primary tumors

^i^ *n* = 514/524 (10 missing)

^j^ *n* = 511/524 (13 missing)

^k^ *n* = 516/524 (8 missing)

^*^ Significant at *p* < .05. ** Significant at *p* < .01. *** Significant at *p* < .001

*n* Sample size, *SD* Standard deviation, *SBSQ-D* Set of Brief Screening Questions-Dutch, *SNS* Subjective Numeracy Scale, *GHS* Global Health Status from the EORTC-QLQ-C30, *EORTC-QLQ-C30* European Organization for Research and Treatment of Cancer Quality of Life Questionnaire for Cancer, *MAC* Mental Adjustment to Cancer, *LOT-R* Life Orientation Test-Revised, *STAI* Spielberger State and Trait Anxiety Inventory, *UCL* Utrecht Coping List, *TFA* Tolerance for Ambiguity, *TiOS-SF* Trust in Oncologist Scale-Short Form

### Patients’ prognostic information preferences and underlying reasons

**Fig. 1:**
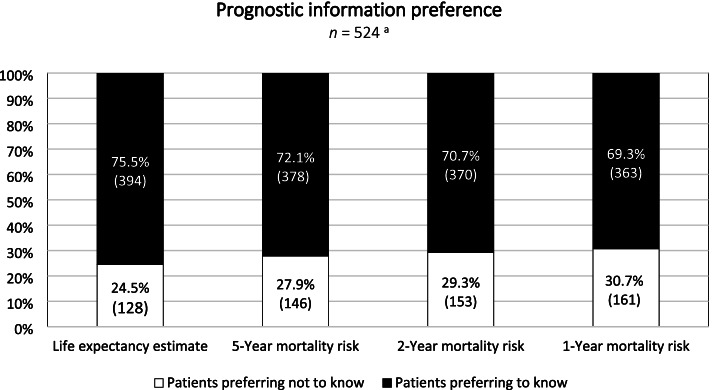
Patients’ preferences for information about a life expectancy estimate and the 5/2/1-year mortality risk. ^a^ *n* = 522/524 patients reported their preference to know a general life expectancy estimate (2 missing) and *n* = 523/524 patients reported their preference to know the 2-year mortality risk (1 missing). Abbreviations: *n*: sample size

One-fourth of patients (25%, *n* = 128/522) preferred not to know a general life expectancy estimate. The proportion of patients preferring unawareness of the mortality risk numerically increased as the indicated period shortened (5/2/1-year); 31% preferred not to know the 1-year mortality risk (Fig. 1).

Patients preferring not to know the 1-year mortality risk showed significantly stronger agreement with most reasons for wanting limited information than patients preferring to know (i.e., staying optimistic, avoiding frightful information, feeling unable to comprehend information). Overall, patients preferring not to know the 1-year mortality risk agreed most strongly with wanting limited information to stay optimistic. The total sample least endorsed “not wanting to be a burden to the physician” as a reason to prefer limited information (Table 3).

**Table 3:** Reasons for wanting limited or complete information about disease and treatment for patients with and without a preference to know prognosis

Reasons	Cronbach’s alpha ^a^	Patients preferring not to know 1-year mortality risk ^b^ *n* = 161/524 mean ± SD	Patients preferring to know 1-year mortality risk *n* = 363/524 mean ± SD
Reasons for wanting **limited** information			
Staying optimistic (CCCI, 1–5) ^c^	.87	3.4 ± 1.1***	2.3 ± 1.1
Avoiding frightful information (CCCI, 1–5) ^c^	.89	2.5 ± 1.1***	1.8 ± 0.9
Feeling unable to comprehend information (CCCI, 1–5) ^d^	.85	1.9 ± 0.9*	1.7 ± 0.9
Not wanting to be a burden to the physician (CCCI, 1–5) ^c^	.89	1.6 ± 0.8	1.6 ± 0.9
Reasons for wanting **complete** information			
Gaining a sense of control (CCCI, 1–5) ^e^	.86	3.4 ± 1.1	4.2 ± 0.9***
Meeting expectations of others (CCCI, 1–5) ^f^	.79	2.1 ± 1.1	2.4 ± 1.3**
Reducing anxiety (CCCI, 1–5) ^e^	.85	2.5 ± 1.1	2.9 ± 1.3***
Gaining autonomy (CCCI, 1–5) ^f^	.71	2.6 ± 1.0	3.1 ± 1.1***

^a^ Interpretation: < 0.50 unacceptable, 0.50–0.60 poor, 0.60–0.70 questionable, 0.70–0.80 acceptable, 0.80–0.90 good, 0.90–1.00 excellent

^b^ Patients preferring not to know agreed more strongly with reasons for wanting complete information than with reasons for wanting limited information (MD = .33; *p* = .001)

^c^ *n* = 522/524 (2 missing)

^d^* n* = 521/524 (3 missing)

^e^* n* = 518/524 (6 missing)

^f^ *n* = 517/524 (7 missing)

^*^ Significant at *p* < .05. ** Significant at *p* < .01. *** Significant at *p* < .001

*SD* Standard deviation, *CCCI* Considerations Concerning Cancer Information Questionnaire, *MD* Mean difference

Contrastingly, patients preferring not to know the 1-year mortality risk showed significantly less agreement with reasons for wanting complete information than patients preferring to know (i.e., gaining a sense of control, meeting expectations of others, reducing anxiety, gaining autonomy). Remarkably, patients preferring not to know prognosis agreed significantly more strongly with reasons for wanting *complete* information than with reasons for wanting *limited* information (Table 3).

### Characteristics related to patients’ prognostic information preference

Univariate tests showed that females compared to males, and low educated compared to high-educated patients, were significantly more likely to prefer not knowing the 1-year mortality risk. Patients receiving a second or ≥ third line of systemic treatment at the time of study participation were more likely to prefer unawareness than patients without systemic treatment. Similarly, patients receiving a ≥ third line of systemic treatment at the time of study participation were significantly more likely to prefer unawareness than patients receiving a first line. Patients perceiving the likelihood of dying within one year as extremely unlikely were more likely to prefer not knowing the 1-year mortality risk than patients perceiving this chance as more likely. The same holds for patients perceiving the likelihood of dying within one year as possible compared to patients perceiving this chance as extremely likely. Patients preferring not to know the 1-year mortality risk had significantly lower numeracy skills, better health-related quality of life, less trait anxiety and stronger fighting spirit than patients preferring to know (Table 2). Patients’ age, nationality, religion, presence of children < 18 years, health literacy, tumor type, time since diagnosis, trait optimism, avoidance coping, uncertainty tolerance and trust in the physician were not related to information preference regarding the 1-year mortality risk.

In the multivariate analysis, females (OR = 1.67, 95%CI [1.12; 2.48], *p* < 0.05) were significantly more likely to prefer not knowing the 1-year mortality risk than males. Patients receiving a ≥ third line of systemic treatment at the time of study participation were significantly more likely to prefer not knowing the 1-year mortality risk than patients receiving a first line (OR = 0.499, 95%CI [0.275; 0.906], *p* < 0.05) or without systemic treatment (OR = 0.375, 95%CI [0.190; 0.742], *p* < 0.01). Additionally, patients with stronger fighting spirit (OR = 1.22, 95%CI [1.13; 1.33], *p* < 0.001) were more likely to prefer not knowing the 1-year mortality risk (Table 4).

**Table 4:** Logistic regression model with predictors of patients’ preference not to know prognosis^a^

	**Final model** ^b c^						
**Predictor**	**B**	**SE**	**Wald**	***p***	**Exp(b)**	**Lower CI**	**Upper CI**
Constant	-2.869	.559	26.390	.000***	.057		
Sex	.511	.203	6.361	.012*	1.667	1.121	2.480
Line of systemic treatment during study participation							
None	-.980	.347	7.958	.005**	.375	.190	.742
First line	-.694	.304	5.225	.022*	.499	.275	.906
Second line	-.154	.336	.210	.647	.857	.444	1.656
≥ Third line (ref)			12.072	.007**			
Fighting spirit (mini-MAC)	.202	.041	24.725	.000***	1.224	1.130	1.326

^a^ 0 = preferring to know the 1-year mortality risk, 1 = preferring not to know the 1-year mortality risk

^b^* n* = 505/524 (19 missing)

^c^ Multilevel analysis was not required, since accounting for clustering within physicians by adding a level did not significantly improve model fit (*p* > .05) and the intraclass correlation was low (0.05). Intercorrelations between predictors were *r* < .60. Patients’ age, nationality, religion, presence of children < 18 years, health literacy, numeracy, tumor type, time since diagnosis, trait optimism, trait anxiety, avoidance coping, uncertainty tolerance and trust in the physician were omitted from the model (*p* > .20). To simplify the final model, educational level, patients’ estimation of the likelihood of dying within one year and health-related quality of life (*p* > .05) were eliminated

^*^ Significant at *p* < .05. ** Significant at *p* < .01. *** Significant at *p* < .001

*B* Unstandardized coefficient, *SE* Standard error, *p* significance, *Exp(b)* exponentiation of the B coefficient, which is an odds ratio, *CI 95%* 95% Confidence Interval, *ref* Reference category, *MAC* Mental Adjustment to Cancer

### Concordance between physicians’ perceived and patients’ actual prognostic information preference

Physicians’ perceptions of and patients’ actual preference for information about the 1-year mortality risk corresponded in 55% of cases (*n* = 285/518) (Table 5). Among patients preferring not to know the 1-year mortality risk, 50% (*n* = 81/161) had a treating physician who accurately reported their information preference; among patients preferring to know, this was 57% (*n* = 204/357). The calculated kappa value of 0.066 suggests poor concordance between physicians and patients.

**Table 5:** Concordance between physicians’ perceived and patients’ actual preference to know prognosis

Physicians’ perceived information preference ^a^	Patients preferring not to know 1-year mortality risk % (*n*)	Patients preferring to know 1-year mortality risk % (*n*)	Total
Patient prefers not to know	50.3 (81) ^b^	42.9 (153) ^c^	234
Patient prefers to know	49.7 (80) ^c^	57.1 (204) ^b^	284
Total	100 (161)	100 (357)	518

^a^ *n* = 518/524 (6 missing)

^b^ Concordance between physicians’ perceived and patients’ actual preference for information about the 1-year mortality risk

^c^ Discordance between physicians’ perceived and patients’ actual preference for information about the 1-year mortality risk

*n* Sample size

## Discussion

### Main findings

We found that 25% of patients prefer not to know a general life expectancy estimate, increasing up to 31% as the indicated period shortens (5/2/1-year). Our univariate results indicate that patients with difficulties in understanding medical information and those feeling relatively well may be more likely to prefer prognostic unawareness. Moreover, psychological factors likely underlie patients’ information preference, given the lower levels of anxiety and stronger fighting spirit among patients preferring not to know. Besides fighting spirit, female sex and a further line of systemic treatment were associated with preferring prognostic unawareness in univariate and multivariate analyses. Importantly, physicians often did not know patients’ prognostic information preference.

### What this study adds

This study is the first to reveal the association between fighting spirit and patients’ prognostic information preference. Perhaps, patients with strong fighting spirit benefit from unawareness, as it enables them to keep hope and push through [28]. Congruently, patients preferring not to know prognosis more often reported optimism, known to relate to fighting spirit, as a reason for wanting limited information [67]. Still, physicians should evaluate the adaptiveness of patients’ prognostic unawareness based on strong fighting spirit, since it may obstruct anticipation of the end-of-life [9, 68]. Considering that patients’ readiness for prognostic discussions could evolve over time, physicians may need to explore patients’ prognostic information preferences repeatedly (e.g., “Some people like to know everything about their illness and what may happen in the future, others prefer not to know too many details. How much would you like to know about your prognosis right now?”; “With regards to your prognosis, have I given you the information you need so far?”) [69–72]. While a patient’s unchanged preference for unawareness should be respected, physicians can consider negotiating for limited prognostic disclosure to assure informed decisions about the best possible (future) care [24, 69]. Another approach is to discuss planning for *hypothetical* deterioration, hereby gaining insight into patients’ wishes without disclosing prognostic estimates [73].

Interestingly, we observed that patients who prefer not to know prognosis generally showed stronger agreement with reasons for wanting complete *versus* limited information. Apparently, patients who prefer prognostic unawareness recognize the relevance of acquiring medical information to, for example, gain a sense of control. This result might exhibit ambivalent attitudes towards prognostic communication, as patients often struggle between wanting clarity and needing hope [16, 24, 74, 75]. The observed ambivalence seems to discourage dichotomization of information preferences (yes/no). Hence, physicians may explain the various types and formats of prognostic information they can offer (e.g., life expectancy, mortality risk, likelihood of experiencing events; point estimates, time frames, multiple scenarios; words, numbers) [14, 22, 24, 71, 76].

We found that females were more likely to prefer not knowing prognosis than males. Conflictingly, previous research shows that females are more likely to report having discussed prognosis and to understand their disease stage, which demands further research on sex differences [77, 78]. In our study, females’ preference for prognostic unawareness might be (partially) attributed to confounding variables, as females were significantly more often low-educated, had lower numeracy skills, were more likely to receive a further line of systemic treatment and reported better health-related quality of life than males. Reviewing the association between patients’ ongoing line of systemic treatment and preference not to know prognosis, we observed that patients receiving a further line relatively more often believed that dying within one year was unlikely. Still, the causal direction of these relations remains unclear.

The concordance between physicians’ perceived and patients’ actual prognostic information preference was nearly the same as would be expected by chance, emphasizing the importance of explicit assessment of individuals’ prognostic information preferences [22, 25, 47]. Possibly, physicians did not standardly explore patients’ information needs, which could relate to reluctance towards prognostic conversations [25, 37, 47, 79–81]. However, we must interpret the poor concordance cautiously. Physician–patient contact may have been limited, as some patients were consulted by multiple physicians and participation was not linked to a specific moment in the disease trajectory. Besides, physicians were unable to report not knowing patients’ preference.

### Strengths and limitations

Firstly, a selection bias potentially occurred as patients who strongly avoid threatening information might have been more likely to decline participation, which could cause an underestimation of the proportion of patients preferring not to know prognosis. Moreover, the study’s generalizability is limited to the Dutch population, which is Western, largely non-religious and known for its straightforwardness. Hence, the wish not to know prognosis might be even more pronounced in other countries. Our sample is also relatively young and certain tumor types are underrepresented. Both age and tumor type were however unrelated to patients’ prognostic information preference. Another limitation concerns the inclusion of a larger sample than planned, which may have led to statistically significant findings that lack clinical relevance. As a definition of minimal clinically important differences in this study’s setting is missing, drawing conclusions about clinical relevance is complicated regardless of sample size. Lastly, the validity and reliability of non-standardized survey items are unknown, and our cross-sectional dichotomous measurement of prognostic information preferences paints a limited picture. Future research could examine changes in patients’ prognostic information preferences over time and investigate the impact of prognostic disclosure on patients who prefer unawareness. Furthermore, there is a need for evidence-based clinical guidelines about how to explore patients’ prognostic information preferences and discuss prognosis effectively. Strengths pertain to the measurement of patients’ preferences regarding different types of prognostic information, and physicians’ perceptions hereof, among a relatively large sample of advanced cancer patients.

## Conclusions

We showed that, in a Western secularized country, a substantial proportion of patients prefer not to know prognosis. This points out the universality of a wish for prognostic unawareness. Although the underlying reasons vary and could be ambivalent, patients’ preference not to know prognosis seems mainly motivated by a need for optimism. Concordance between physicians’ perception of and patients’ actual prognostic information preference was poor. We encourage physicians to assess patients’ prognostic information preferences and explore their motivations explicitly and repeatedly, rather than making assumptions based on patient characteristics shown to relate to prognostic information preferences [14, 71, 82]. Physicians can explain the variety in type and detail of prognostic information they can offer to promote individually tailored communication [71].

## Supplementary Information

**Additional file 1.** Overview of eligible tumor types, specified by non-treated and treated cancer. This overview is not inclusive. Physicians could include patients with other tumor types, for whom the general inclusion criteria were applicable (≥18 years, Dutch language proficiency, diagnosis of metastatic or locally inoperable cancer at least two months before participation, not eligible for therapy with curative intent, median survival of 12 months or less on group-level). Patients could participate when receiving anticancer therapy or comfort care.

**Additional file 2.** Flowchart showing inclusion, exclusion and response of the PROSPECT study.

## Data Availability

The datasets analyzed during the current study are not publicly available due to ethical considerations, but are available from the corresponding author on reasonable request. Additional files for this article, including a flowchart showing inclusion, exclusion and response of the PROSPECT study and an overview of eligible tumor types (specified by non-treated and treated cancer) is available online.

## Abbreviations

B: Unstandardized coefficient
CCCI: Considerations Concerning Cancer Information Questionnaire
CI95%: 95% Confidence Interval
EORTC-QLQ-C30: European Organization for Research and Treatment of Cancer Quality of Life Questionnaire for Cancer
Exp(b): Exponentiation of the B coefficient, which is an odds ratio
GHS: Global Health Status
LOT-R: Life Orientation Test-Revised
MAC: Mental Adjustment to Cancer
MD: Mean difference
*n*: Sample size
OR: Odds ratio
*p*: Significance
Ref: Reference category
SBSQ-D: Set of Brief Screening Questions-Dutch
SD: Standard deviation
SE: Standard error
SNS: Subjective Numeracy Scale
STAI: Spielberger State and Trait Anxiety Inventory
TFA: Tolerance for Ambiguity
TiOS-SF: Trust in Oncologist Scale-Short Form
UCL: Utrecht Coping List
