# Supplementary material for: Characteristics of patients with advanced cancer preferring not to know prognosis: a multicenter survey study
Source: BMC Cancer. 2022 Sep 1;22:941. doi: 10.1186/s12885-022-09911-8 (PMC9434918; doi:10.1186/s12885-022-09911-8)
Supplement: Supplementary file 1 — Additional file 1. Overview of eligible tumor types, specified by non-treated and treated cancer. This overview is not inclusive. Physicians could include patients with other tumor types, for whom the general inclusion criteria were applicable (≥18 years, Dutch language proficiency, diagnosis of metastatic or locally inoperable cancer at least two months before participation, not eligible for therapy with curative intent, median survival of 12 months or less on group-level). Patients could participate when receiving anticancer therapy or comfort care. [file 12885_2022_9911_MOESM1_ESM.docx]

**Additional files**

**Additional file 1.** Overview of eligible tumor types, specified by non-treated and treated cancer. This overview is not inclusive. Physicians could include patients with other tumor types, for whom the general inclusion criteria were applicable (≥18 years, Dutch language proficiency, diagnosis of metastatic or locally inoperable cancer at least two months before participation, not eligible for therapy with curative intent, median survival of 12 months or less on group-level). Patients could participate when receiving anticancer therapy or comfort care.

| **Absence of disease-oriented therapy:** | | **Presence of disease-oriented therapy, inclusion in case of:** |
| --- | --- | --- |
| Stage IV | Liver cancer | All lines of systemic therapy |
|  | Biliary tract cancer | All lines of systemic therapy |
|  | Gall bladder cancer | All lines of systemic therapy |
|  | Pancreatic cancer | All lines of systemic therapy |
|  | Sarcoma | All lines of systemic therapy |
|  | Endometrium cancer | All lines of systemic therapy |
|  | Cervix cancer | All lines of systemic therapy |
|  | Unknown primary | All lines of systemic therapy |
|  | Stomach cancer | All lines of systemic therapy, *except* first line trastuzumab |
|  | Oesophagogastric cancer | All lines of systemic therapy, *except* first line trastuzumab |
|  | Ovarian cancer | Platinum resistant disease |
|  | Prostate cancer | Second generation anti-androgen resistant disease *or* progression after the last line cabazitaxel |
|  | Breast cancer | Fourth line of systemic therapy *or* triple negative disease |
|  | Bladder cancer | Second line of systemic therapy |
|  | Colorectal cancer | Third line of systemic therapy |
|  | Anal cancer | Progression after the first line of systemic therapy |
|  | Renal cell cancer | Third line of systemic therapy for MSKCC intermediate *or* poor risk |
|  | Neuroendocrine carcinoma | Second line of systemic therapy |
|  | Small cell lung cancer | All lines of systemic therapy |
|  | Non-small cell lung cancer without driver mutation | Second line of immuno(chemo)therapy *or* all lines of chemotherapy |
|  | Non-small cell lung cancer with driver mutation | Last line of systemic therapy |
|  | Mesothelioma | All lines of systemic therapy |
|  | Thymoma | Second line of systemic therapy |
| Grade IV | Glioblastoma | Progression after chemo-radiation |
