# Supplementary material for: Characteristics of patients with advanced cancer preferring not to know prognosis: a multicenter survey study
Source: BMC Cancer. 2022 Sep 1;22:941. doi: 10.1186/s12885-022-09911-8 (PMC9434918; doi:10.1186/s12885-022-09911-8)
Supplement: Supplementary file 2 — Additional file 2. Flowchart showing inclusion, exclusion and response of the PROSPECT study. [file 12885_2022_9911_MOESM2_ESM.docx]

**Additional file 2.** Flowchart showing inclusion, exclusion and response of the PROSPECT study.


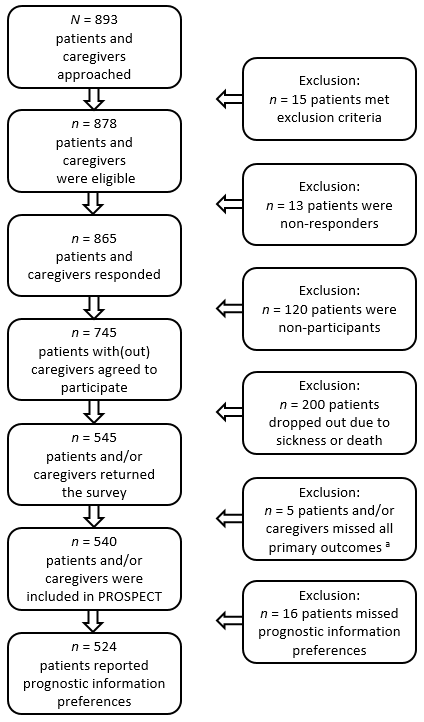


^a^ Primary outcomes of PROSPECT were prognostic information preferences and prognostic awareness.
